# Review of Antibiotic Resistance in the Indian Ocean Commission: A Human and Animal Health Issue

**DOI:** 10.3389/fpubh.2017.00162

**Published:** 2017-07-06

**Authors:** Noellie Gay, Olivier Belmonte, Jean-Marc Collard, Mohamed Halifa, Mohammad Iqbal Issack, Saindou Mindjae, Philippe Palmyre, Abdul Aziz Ibrahim, Harena Rasamoelina, Loïc Flachet, Laurent Filleul, Eric Cardinale

**Affiliations:** ^1^Animals, Health, Territories, Risks and Ecosystems Unit, Department of Animal Health, French Agricultural Research Center for International Development (CIRAD), Montpellier, France; ^2^Bacteriology Laboratory, Félix Guyon Hospital, Saint-Denis, Reunion; ^3^Experimental Bacteriology Unit, Pasteur Institute of Madagascar, Antananarivo, Madagascar; ^4^El Maroof Hospital, Moroni, Comoros; ^5^Central Health Laboratory, Victoria Hospital, Candos, Mauritius; ^6^General Direction of Health, Moroni, Comoros; ^7^Victoria Hospital, Victoria, Seychelles; ^8^Health Monitoring Unit, Indian Ocean Commission, Port-Louis, Mauritius; ^9^Regional Unit of Indian Ocean, Santé Publique France, Saint-Denis, Reunion

**Keywords:** Indian Ocean, epidemiology, antimicrobial resistance, One Health, prevalence, surveillance, zoonosis

## Abstract

Antimicrobial resistance (AMR) is a major threat to human, animal health, and environment worldwide. For human, transmission occurred through a variety of routes both in health-care settings and community. In animals, AMR was reported in livestock, pets, and wildlife; transmission of AMR can be zoonotic with the probably most important route being foodborne transmission. The Indian Ocean Commission (IOC), composed of Comoros, Madagascar, Mauritius, Reunion (France), and Seychelles recognized the surveillance of AMR in both animal and human as a main public health priority for the region. Mayotte, French overseas territory, located in Comoros archipelago, was also included in this review. This review summarized our best epidemiological knowledge regarding AMR in Indian Ocean. We documented the prevalence, and phenotypic and genotypic profiles of prone to resistance Gram-positive and Gram-negative bacteria both in animals and humans. Our review clearly pointed out extended-spectrum β-lactamase and carbapenemase-producing Enterobacteriaceae as main human and animal health issue in IOC. However, publications on AMR are scarce, particularly in Comoros, Mayotte, and Seychelles. Thus, research and surveillance priorities were recommended (i) estimating the volume of antimicrobial drugs used in livestock and human medicine in the different territories [mainly third generation cephalosporin (3GC)]; (ii) developing a “One Health” surveillance approach with epidemiological indicators as zoonotic foodborne pathogen (i.e., couple *Escherichia coli* resistance to 3GC/carbapenems); (iii) screening travelers with a history of hospitalization and consumption of antibiotic drug returning from at risk areas (e.g., mcr-1 transmission with China or hajj pilgrims) allowing an early warning detection of the emergence for quick control measures implementation in IOC.

## Introduction

Increasing global antimicrobial resistance (AMR) is a major threat to human and animal endangering decades of improvements in health-care outcomes. It endangers modern human and veterinary medicine and undermines food safety (1).

In humans, the global burden of AMR is longer duration of illness, higher lethality, increasing costs of treatment, and inability to cure infected patient (2). In animals, antibiotic drugs use in terrestrial and aquatic animals maintains food safety, animal welfare, and protect livelihoods (3).

Transmission of AMR to human can be zoonotic taking place through a variety of routes where the foodborne route is probably the most important (4). Direct transmission also occurs for specific bacteria species [e.g., methicillin-resistant *Staphylococcus aureus* (MRSA)].

The Indian Ocean Commission (IOC) is composed of five countries: Comoros, Madagascar, Mauritius, Reunion (France), and Seychelles. Mayotte, French overseas territory located in Comoros archipelago, was also included in the study. In 2015, the IOC identified AMR surveillance in both animal and human as a main priority for territories (5). However, AMR burden is not well evaluated but epidemiological trends in IOC should be identified.

Our systematic review objective was summarizing epidemiological knowledge and trends of AMR in prone-to-multidrug resistance bacteria species (6), and fecal–oral and foodborne bacteria in human and animals in IOC. We documented the prevalence, and phenotypic and genotypic profiles of resistance of the selected bacteria in (i) Gram-positive and (ii) Gram-negative bacteria.

## Materials and Methods

The study performed from January to March 2017 included articles and conference abstracts published from 1990 to December 2016. Bacteria species included in the different searches were those prone to develop multidrug resistance as defined by Magiorakos et al. (6) (i.e., *S. aureus, Enterococcus* spp., *Pseudomonas aeruginosa, Acinetobacter* spp., and the genus Enterobacteriaceae), and fecal–oral and foodborne bacterial species (*Salmonella* spp., *Campylobacter* spp., and *Shigella* spp.). We used available articles obtained through match searches using Google Scholar,^1^ PubMed,^2^ and Web of Knowledge.^3^ Relevant information was obtained for phenotypic and genotypic profiles of resistance in selected bacteria using its name combined with related terms (resistance, antimicrobial, antibiotic, Comoros, Seychelles, Reunion Island, Madagascar, Mayotte, Indian Ocean, epidemiology). Included publications were those documenting the prevalence, and phenotypic and genotypic profiles of resistances of selected bacteria; others were excluded.

## Results and Discussion

A total of 42 articles were considered relevant for the review.

### Gram-Positive Bacteria

#### *Staphylococcus* *aureus*

*Staphylococcus aureus* is one of the most common causes of nosocomial and community infections (7). Since 1960s, MRSA was isolated (8) and became a major nosocomial pathogen (9).

In Madagascar, MRSA was increasing from 2001 to 2014 as observed in Table 1 with rising rate of resistance to oxacillin and cefoxitin. MRSA nasal carriage in community was observed with a prevalence of 14.8% in 2011 (10, 11). Overall resistances were higher for widely available drugs (12). Moreover, 9.0% of veterinary students were asymptomatic MRSA carriers (11). Increasing rate of resistance to gentamicin (42.9%) and vancomycin (7.1%) was observed in MRSA isolates (11). Nonetheless, vancomycin and tigecycline are the last drugs demonstrating therapeutic efficacy for MRSA and are compromised by the reduced susceptibility in *S. aureus* (13). Phenotypic resistance observed in Madagascar was of concern (11), confirmation by an antimicrobial reference laboratory for genotyping should be considered.

**Table 1:** Evolution of antibiotic resistance of *S. aureus* from 2001 to 2014 in Indian Ocean Commission.

Country	Population	Year	Study design	Sample type	Isolates number	OXA/CEF	PEN	ERY	LIN	SXT	GEN	Reference
Madagascar	Com	2001–2005	Laboratory surveillance	Pus, genital, urine, respiratory	68	6.5%	87.9%	14.6%	6.1%	16.8%	1.9%	(12)
Madagascar	Hosp	2001–2005	Laboratory surveillance	Surgical wounds, pus, hemoculture	506	4.4%	91.2%	10.3%	7.3%	13.2%	0.0%	(12)
Reunion	Hosp	2007	Laboratory surveillance	Unknown (diagnostic specimen)	–	13%	85.0%	18.0%	11%	0.4%	0.8%	(14)
Madagascar	Hosp	2010	Laboratory surveillance	Surgical wounds, pus, burn, urine, respiratory	103	13.6%	92.2%	19.4%	5.8	NI	3.9%	(15)
Mauritius	Hosp	2010	Laboratory surveillance	Unknown (diagnostic specimen)	127	37.8%	95.3%	27.6%	NI	NI	NI	(16)
Madagascar	Com	2011	Cross-sectional study	Nasal swabs	45	38.8%	100.0%^a^	66.7%^a^	31.1%^a^	68.9%^a^	4.4%^a^	(10)
Madagascar	Com	2011–2013	Laboratory surveillance	Urine	48	8.3%	75.0%	NI	NI	58.3%	NI	(17)
Madagascar	Com (veterinarian)	2013–2014	Cross-sectional study	Nasal swabs	30	46.7%	100%	60.0%	NI	76.7%	20%	(11)
Madagascar	Com (veterinarian)	2013–2014	Cross-sectional study	Nasal swabs	14	100.0%^a^	100%^a^	64.3%^a^	NI	71.4%^a^	42.9%^a^	(11)
Mauritius	Hosp	2014	Laboratory surveillance	Blood culture, pus, burn, urine, swab, respiratory intravascular catheter	140	39.0%	NI	31.0%	NI	NI	NI	(18)

*OXA, oxacillin/CEF, cefoxitin; PEN, penicillin; ERY, erythromycin; LIN, lincomycin; SXT, trimethoprim sulfamethoxazole; GEN, gentamicin; NI, not identified*.

*^a^Resistance in methicillin-resistant Staphylococcus aureus strains*.

In Mauritius, in May 2010, among all *S. aureus* isolated in hospitalized patients’ infections, 37.8% were MRSA (16) and 39% were MRSA in July 2014 (18). All *S. aureus* tested were susceptible to vancomycin (16, 18).

In Reunion, MRSA increased from 16.0% in 1997 to 23.0% in 2000 and decreased to less than 15.0% in 2006/2007 (14). *S. aureus* rate of resistance to all antibiotic drugs decreased from 1997 to 2007, with the exception of the fusidic acid (16.0% in 2007). Since 1998, *S. aureus* susceptibility profile changed tending to be more resistant to aminoglycosides and quinolones (14).

In the other territories, literature remains absent and no publication was found regarding prevalence and antibiotic resistance profiles of *S. aureus* in humans, livestock, and pets.

Methicillin-resistant *Staphylococcus aureus* epidemiological trends should be better addressed in Comoros, Mayotte, Reunion, and Seychelles. Rates of resistances for widely available oral agents observed in Madagascar could point out a drug overuse in this territory as well as in Mauritius.

Burden of MRSA in livestock and pets should be addressed particularly as they can directly contaminate veterinary, breeders, or other animals (19).

#### *Enterococcus* spp.

*Enterococcus* spp. are opportunistic bacteria often involved in nosocomial infections, mainly urinary tract infections, endocarditis, wounds, and bacteremia (20). Discovered by the end of the 1980s (21), vancomycin-resistant enterococci (VRE) represent a major problem in health-care settings worldwide.

In Madagascar, in 2006–2008, rate of resistance to vancomycin in *Enterococcus* spp. was 3.3% and was high for lincomycin (90.0%) (15). In 2011–2013, one *E. faecalis* resistant to vancomycin (5.6%) was isolated during an uropathogenic survey (17).

In Mauritius, in May 2010 and July 2014, all *Enterococcus* spp. isolated in hospitalized patients were susceptible to vancomycin (16, 18).

In Reunion, Picot et al. (22) did not detect any VRE in 2005. In other territories, no publication was found for both humans and animals.

Thus, epidemiological situation of VRE is not clear in IOC but does not seem being a main issue both in animals and humans. Identifying drug uses in livestock remains necessary as glycopeptide (e.g., avoparcin) use in livestock was correlated with VRE incidence in human populations (23).

### Gram-Negative Bacteria

#### *Pseudomonas* *aeruginosa*

*Pseudomonas aeruginosa* is an opportunistic pathogen causing nosocomial infections (24). Some strains have been found to be resistant to nearly all antibiotics (25).

In Madagascar, in 2006–2008, *P. aeruginosa* isolates showed a moderate resistance to penicillin (piperacillin 12.8% and ticarcillin 31.9%) but were susceptible to ceftazidime and imipenem (15).

In Mauritius, in May 2010, *P. aeruginosa* isolated in hospitalized patients showed high rate of resistance to antibiotic tested with 51.5% to aminoglycosides (52% gentamicin and 51% amikacin), 47% for ceftazidime, 73% for ciprofloxacin, and 40% for meropenem (16). In July 2014, a similar survey pointed a decrease of all resistance tested (42% for gentamicin, 29% for amikacin, 30% for ceftazidime, 47% for ciprofloxacin, and 27% for meropenem) (18).

In Reunion, rates of resistance to imipenem increased from 1997 to 2005 (5.9–6.1%) (22). Outbreaks of *P. aeruginosa* were reported in a neonatal care unit (26, 27), but susceptibility testing of strain was not performed. In Reunion, between 2010 and 2012, OXA-221 was identified in *P. aeruginosa* associated with β-lactamases and carbapenemase production (28). The extended-spectrum β-lactamase OXA-145 was described in 2011 in *P. aeruginosa* and conferred resistance to third generation cephalosporin (3GC) and monolactams (29).

Trends regarding *P. aeruginosa* in IOC are not clear, but acquisition of new gene of resistance to β-lactams is of concern. Rate of resistance to carbapenems identified in Mauritius are high even if decreasing. Identifying its burden in nosocomial infection is needed as well as aminoglycoside resistance. Recommended treatment for pseudomonas infection usually includes β-lactams and aminoglycosides.

#### *Acinetobacter* spp.

*Acinetobacter baumannii* is a troublesome pathogen for health-care institutions, and its ability to acquire resistance determinants is making it threatening the current antibiotic era (30).

In Madagascar, in 2006–2008, a prevalence of *A. baumannii* of 8.8% was identified in infections diagnosed at hospital, the resistance to ceftazidime (62.0%) and imipenem was high (45.7%) (15). Among strains collected between 2006 and 2009, 92.5% were resistant to imipenem and 94.3% to ceftazidime (31). No resistance to carbapenems was reported among ten uropathogenic isolates in community (2011–2013) (17), but this restricted sample could not reflect the epidemiological situation. The dissemination of multidrug-resistant OXA-23-producing *A. baumannii* in hospitals of Antananarivo (31) could emphasize issues regarding failures of infection control in hospitals (15).

In Mauritius, in May 2010, *Acinetobacter* spp. isolated in hospitalized patients showed high rate of resistance to antibiotic tested with 86% for gentamicin and 50% for amikacin (both aminoglycosides), 95% for cefotaxime, 85% for ciprofloxacin, and 68% for meropenem (16). In July 2014, a similar survey pointed a decreasing rate of resistance for gentamicin, cefotaxime, and ciprofloxacin (respectively 79, 94, and 82%) and increasing for amikacin (58%) and meropenem (74%) (18).

In Reunion, from 1997 to 2005, *A. baumannii* rate of resistance decreased to all antibiotic tested [e.g., ceftazidime (74.3–68.1%), imipenem (12.9–8.3%), and ciprofloxacin (72.9–59.7%)] (22). In 2006, an outbreak of multiresistant *A. baumannii* phenotype 5 occurred at the hospital, strains were resistant to all β-lactams (32). A wide variety of *A. baumannii* sequence types was identified at the hospital and could be related to community-acquired infections; one isolate carrying the $bla_{{\text{OXA-23}}^{-}}$ gene was identified (33).

In Comoros, the $bla_{{\text{OXA-23}}^{-}}$ gene in *A. baumannii* was identified in 2011 (34).

Pets can be reservoir of *A. baumannii* (35). In Reunion, the prevalence in pets was 8.5% but no isolates were resistant to carbapenems (33). In cattle, the first case of OXA-24^−^ producing *A. baumannii* was recently identified (36).

Resistance to carbapenems in *A. baumannii* was observed in Comoros, Madagascar, Reunion, and Mauritius. It is of concern for IOC as *A. baumannii* have an affinity with vulnerable patients (37). Producing-carbapenemase *A. baumannii*, with the dissemination of OXA-23 enzymes, should be thoroughly monitored, keeping in mind the possible clonal spread of multiresistant strains in hospital, community, and pets.

#### Enterobacteriaceae

β-Lactamase production are the primary cause of resistance among members of the family Enterobacteriaceae. In recent years, β-lactamases have extensively diversified in response to clinical use of β-Lactams (38).

##### Extended-Spectrum β-Lactamase-Producing Enterobacteriaceae (ESBLE)

Extended-spectrum β-lactamase-producing Enterobacteriaceae confer resistance to β-lactam antibiotics except cephamycins and carbapenems and are inhibited by clavulanic acid (39).

Extended-spectrum β-lactamase-producing Enterobacteriaceae was first isolated in Madagascar between 2004 and 2006 in urinary tract infections (40) as observed in Table 2. High fecal carriage of ESBLE was identified in both hospital and community with prevalence of 21.3% in two hospitals from 2006 to 2008 (15), 21.2% in a pediatric hospital in 2008 (41), and 10.1% in community in 2009 (42). Between 2013 and 2014, 18.5% of rectal colonization was estimated among pregnant women at delivery (43). Another study (2015) pointed out 7.1% of Enterobacteriaceae nasal carriage resistance to 3GC in patients at admission (44). A study conducted from 2012 to 2013 pointed out the burden of ESBLE in early neonatal infection (12.9%); infections were treated with expanded spectrum cephalosporins due to the lack of carbapenems and resulted in a high lethality (45%) (45). In Madagascar, ESBLE mostly belong to the CTX-M-15 type (45, 46), widely distributed worldwide (47).

**Table 2:** Evolution of antibiotic resistance of Enterobacteriaceae from 2004 to 2013 in Indian Ocean Commission.

Country/year	Population	Study design	Sample type	Isolates number	ESBL carriers/individuals tested	ESBLE/Enterobacteriaceae tested	AMX	AMC	CAZ/CEF	GEN	NAL	CIP	SXT	Bacterial species	Reference
Madagascar 2004–2006	Com	Laboratory surveillance	Urine	775	NI	3.8%	76.4%	15.6%	4.0%/-	9.2%	24.5%	15.4%	64.8%	*Escherichia coli, Klebsiella pneumoniae, Proteus spp., Enterobacter spp., Citrobacter spp*.	(40)
Mauritius 2005	Com	Laboratory surveillance	Urine	224	NI	12.9%	NI	1.60%	-/9.0%	9.9%	34.0%	26.4%	49.5%	*E. coli, Klebsiella spp., Proteus spp*.	(48)
Reunion 2006–2007	Hosp	Laboratory surveillance	Unknown (diagnostic specimen)	240^a^	NI	5.8%	NI	NI	NI	67.0%	NI	74.0%	75.0%	*E. coli, Enterobacter cloacae, K. pneumoniae*	(32)
Madagascar 2006–2008	Hosp	Laboratory surveillance	Surgical wounds, pus, burn, urine, respiratory	249	NI	21.3%	91.0%	69.0%	26.0%/26.0%	31.0%	52.0%	41.0%	71.0%	*E. coli, K. pneumoniae, Proteus spp., Enterobacter spp., Citrobacter spp*.	(15)
Madagascar 2008	Hosp	Cohort study	Stool	30^a^	57.10%	NI	100.0%	100.0%	86.2%	91.4%	62.0%	50.0%	96.5%	*E. coli, K. pneumoniae*	(41)
Madagascar 2008	Com	Cohort study	Stool	58^a^	22.10%	NI	100.0%	100.0%	90.0%	76.7%	63.3%	46.7%	93.3%	*E. coli, K. pneumoniae*	(41)
Madagascar 2008–2009	Com	Cross-sectional study	Stool	195	NI	3.1%	82.1%	1.0%	1.5%/3.1%	1.0%	10.8%	3.1%	84.6%	*E. coli*	(49)
Madagascar 2009	Com	Cross-sectional study	Stool	53^a^	NI	NI	100.0%	98.0%	NI	NI	68.6%	60.8%	90.2%	*E. coli, K. pneumoniae, Enterobacter spp., Citrobacter spp*.	(42)
Mauritius 2010	Hosp	Laboratory surveillance	Unknown (diagnostic specimen)	195	NI	NI	NI	NI	46.7%	50.6%	NI	39.2%	NI	*E. coli, K. pneumoniae*	(16)
Madagascar 2011–2013	Com	Laboratory surveillance	Urine	224^a^	NI	33.0%	80.8%	58.0%	30.4%- 30.4%	NI	NI	NI	69.2%	*E. coli, Citrobacter spp., K. pneumoniae, Proteus spp., Serratia spp*.	(17)
Madagascar 2013–2014	Pregnant women	Cohort study	Stool	66^a^	18.5%	NI	NI	NI	NI	NI	NI	36.0%	NI	*E. coli, Citrobacter freundii, K pneumoniae, Enterobacter cloacae, Morganella morganii*	(43)
Mauritius 2014	Hosp	Laboratory surveillance	Blood culture, pus, burn, urine, swab, respiratory intravascular catheter	301	NI	NI	NI	NI	50.7%	33.2%	NI	56.1%	NI	*E. coli, K. pneumoniae*	(18)

*AMX, amoxicillin; AMC, amoxicillin + claviculanic acid; CAZ, ceftazidime; SXT, trimethoprim sulfamethoxazole; GEN, gentamicin; CIP, ciprofloxacin; NAL, nalidixic acid; CEF, cefotaxim; NI, not identified; ESBLE, Extended-Spectrum β-Lactamase-Producing Enterobacteriaceae*.

*^a^ESBL isolates only*.

In Mauritius, in 2005, rate of resistance to 3GC in Enterobacteriaceae from urine of patients with presumed community-acquired infection was about 9.0% for cefotaxime and 14.7% for cefixime (Table 2). Isolates also showed high rates of resistance to fluoroquinolones (26.4% to ciprofloxacin) (48). Between 2010 and 2014, an increase of resistance in Enterobacteriaceae isolated in hospitalized patients was observed (46.7–50.7% for cefotaxime and 39.2–56.1% for ciprofloxacin) (16, 18).

In Reunion, prevalence of ESBLE was increasing at hospital with 2.0% in 1997 and 5.8% in 2007 (32). Broad spectrum antibiotics use in hospitals was likely correlated with this evolution (32). In 2013, main ESBLE involved in infections were *Klebsiella pneumoniae* (38.0%), *Escherichia coli* (37.0%), and *Enterobacter cloacae* (24.0%) with 75.0% of the CTX-M-15 type (50).

A prevalence of 14.5% ESBLE was estimated among dogs and cats from veterinary clinics of Reunion Island (51).

In livestock from Comoros, Madagascar, Mauritius, and Mayotte, overall 22.7% of livestock sampled were ESBLE carriers. The highest prevalence was observed among pigs (42.0%) and poultry (26%); main contaminated farms were located in Madagascar (40.5%), Mayotte (26.9%) followed by Mauritius (13.5%) and Comoros (6.7%) (52).

No publication was found for Seychelles, but prevalence of ESBLE (mainly represented by *E. coli, K. pneumoniae*, and *E. cloacae*) in IOC seems increasing both in humans and animals. Broad spectrum antibiotics overuse is likely correlated with this evolution (32).

##### Carbapenemase-Producing Enterobacteriaceae (CPE)

In Madagascar, first CPE was reported in a community survey of uropathogens implemented in 2011–2013 (17). Imipenem rate of resistance was 40.0% for *K. pneumoniae*, 15.0% for *E. cloacae*, and 2.3% for *E. coli* (17). The reduced sample size for this study could not reflect global resistances patterns.

In Mauritius, rate of resistance to meropenem in Enterobacteriaceae increased from 0.51% in 2010 to 5.32% in 2014 among hospitalized patients (16, 18).

In Reunion, in 2007, resistance to imipenem in Enterobactericeae was low (1 to 2 cases by year) (14).

First detection of New Delhi metallo-β-lactamase-1 (NDM-1) gene in *K. pneumoniae* in IOC occurred in a Mauritius patient in 2009 (53), in 2011 in Reunion (54), and in 2013–2014 in Madagascar (43).

In Mayotte, an outbreak of CPE involving *E. cloacae* of IMI-1 type occurred at the hospital (55). First investigations tend to highlight a community source of contamination but further investigations should confirm it (55).

In animal, no publication was found regarding CPE.

Carbapenemase-producing Enterobacteriaceae are endangering the ability to cure infectious diseases. NDM-1 fast propagation in IOC is pointing the need of AMR surveillance and alert system. Mauritius seems particularly affected by CPE; their spread could constitute an issue for other territories as few therapeutic alternatives are available to treat infected patients.

##### Foodborne and Fecal–Oral Origin Enterobacteriaceae

###### *Non-Typhoidal Salmonella* spp.

Salmonella is a major foodborne pathogen found worldwide. Most human salmonellosis is associated with eating contaminated raw or undercooked chicken, eggs, pork, and contaminated water.

In Madagascar, in 2008–2009, *Salmonella* spp. rate of resistance in community children was low for 3GC (1.2% for ceftazidime and cefotaxime), absent for quinolones, and moderated for ampicillin (35.7%) and ticarcillin (35.7%) (49).

In Mauritius, in 2009, *Salmonella enterica* serovar Enteritidis was isolated in humans without resistance identified for all antibiotic tested; transmission by chicken consumption was suspected (56). In 2014, *Salmonella* spp. isolated in stools of patients with gastroenteritis were all sensitive to ampicillin and ciprofloxacin and presented low resistance to trimethoprim–sulfamethoxazole (2%) and nalidixic acid (1%) (57).

In Reunion, between 2007 and 2009, rates of resistance among *Salmonella* spp. isolated in broiler chicken flocks were of 38.3% to streptomycin, 31.8% to tetracycline, and 16.8% to ampicillin (58), but no resistance to 3GC was identified. *S. Hadar* displayed reduced susceptibility to fluoroquinolones (80.8% to enrofloxacin) (58).

In Comoros, no resistance in *Salmonella* spp. was identified between 1987 and 1988 (59).

Publications regarding AMR among *Salmonella* spp. are scarce in IOC. Resistances to quinolones in *Salmonella* spp. seems appearing in Reunion, probably do to its overuse, but not in Mauritius.

###### *Campylobacter* spp.

*Campylobacter* spp. can cause both gastroenteritis and extra-intestinal disease. *C. jejuni* and *C. coli* are the most often isolated from patients with diarrhea as confirmed in Madagascar in 2010–2012 (70.1 and 23.6%, respectively) (60). Main infection source in humans is undercooked chicken, raw or unpasteurized milk, and cross-contamination from the environment (61).

In Madagascar, rate of resistance in *Campylobacter* spp. was moderate in community children in 2008–2009, with overall resistance of 24.8% for amoxicillin, 2.2% for ciprofloxacin, 1.8% for erythromycin, and 1.1% for tetracycline (49). Rate of resistance was higher for *C. coli* (49).

In animals, *Campylobacter* spp. collected in 2005–2006 from chicken neck skin in Madagascar presented 35.8% of resistance to ampicillin, 18.3% to erythromycin, 5.5% to ciprofloxacin, and 3.7% to nalidixic acid (62).

In Mauritius, in 2014, *Campylobacter* spp. isolated in gastroenteritis cases presented high resistance to quinolones with 51% of resistance to ciprofloxacin and 4% to erythromycin (57). High quinolone resistance in *Campylobacter* spp. is probably due to antibiotic overuse in veterinary medicine (57).

Publications regarding AMR among *Campylobacter* spp. are scarce in IOC particularly in animals. Resistance to quinolones in Mauritius could be due to its overuse in poultry industry (57).

###### *Shigella* spp.

*Shigella* spp. is responsible for dysentery predominating in developing countries (63).

In Madagascar, in 1988–1989, resistances in *Shigella dysenteriae* started being observed (64). In 2008–2009, rate of resistance in community children were high for widely used drugs (e.g., 79.9% for trimethoprim-sulfamethoxazole, 62.8% for amoxicillin, 62.2% for ticarcillin) but no resistance for ciprofloxacin was reported (49).

In Comoros, *Shigella* spp. isolated between 1987 and 1988 did not exhibit significant resistances (59).

Few up-to-date publications regarding AMR in *Shigella* spp. were found.

## Perspectives

One main challenge regarding this review was the data collection and comparison of AMR patterns between territories in the diversity of study designs (diagnosis isolates vs. systematic detection), antibiogram panels, over different periods of time and targeting various bacteria species. Thus, results should be interpreted with caution but this attempt of review was not performed before and confirmed that AMR is threatening IOC. Main issue identified for IOC was ESBL and CPE which is in agreement with their increase worldwide over the past decade (65).

Literature was limited in Comoros, Mayotte, and Seychelles confirming needs to develop AMR surveillance and research in these territories and scarce for bacterial species: *Enterococcus* spp., *P. aeruginosa, Salmonella* spp., *Campylobacter* spp., and *Shigella* spp.

In IOC, the SEGA–One Health network was created in 2009 with the objective of monitoring outbreak-prone infections (66). It aims to develop a surveillance of AMR in human and animals but disparity of resources between territories and between animal and human health could be a brake in the establishment of such a system.

Thus, priorities should be established:

(i) A direct relationship between antibiotic consumption, emergence, and dissemination of AMR was demonstrated (67). Estimating the volume of antimicrobial drugs used, types, and access (e.g., over-the-counter) is an essential step for IOC. The overuse of 3GC could have driven to selection pressures on bacterial community in both animals and humans observed (i.e., ESBLE and CPE in hospitals with carbapenems use). Drugs monitoring could help predicting risks of emergence in territories (68). Research on drug uses and habits in community, by practitioners, and in livestock should be explored to adapt control measures.
(ii) Integrated AMR One Health approach including human, animal, and environment in both surveillance and research is clearly needed (e.g., MRSA in veterinarians, *A. baumannii* in pets, humans, and livestock, and ESBLE in livestock and humans). Using standardized indicators (antibiotic drugs–bacteria species couple) for surveillance of AMR patterns across health-care settings, countries, and host species is essential. One relevant epidemiological indicator, for both animal and human, could be *E. coli* AMR, particularly 3GC and carbapenems. Surveillance should be accompanied by further investigations regarding genetic support of resistance between hosts for source of contamination and dynamics of propagation between reservoirs identification (human, animal, and environment).
(iii) Spread of NDM-1-producing Enterobacteriaceae in IOC confirms needs for strengthening the early warning surveillance system of AMR emergences. The region is connected to hotspots of AMR as Asia (12.0% of traffic) characterized by high AMR prevalence in community [e.g., ESBL in China (69), important antibiotic consumption (70), and emergence of new AMR profiles (e.g., NDM-1, mcr-1) (71)]. Screening of travelers, returning from at risk AMR areas, with a history of hospitalization and consumption of antibiotic drugs abroad has been recently proposed (72) and could be relevant for an early emergence detection. However, screening is costly, thus, initiating reflection regarding pooling laboratory resources is essential.

Our article is the first attempt summarizing knowledge regarding AMR in both animal and human health sectors in IOC. This review clearly points out research and surveillance gaps and constitutes a tool for future activities to lead.

## Author Contributions

NG performed the review, collected the data from literature, and wrote the first draft of the manuscript; EC, OB, and LFilleul revised and provided first feedback for the manuscript. All authors provided articles, have drafted, and revised the work critically for important intellectual contents and approved the final version. All authors agreed to be accountable for all aspects of the work in ensuring that questions related to the accuracy or integrity of any part of the work are appropriately investigated and resolved.

## Conflict of Interest Statement

The authors declare that the research was conducted in the absence of any commercial or financial relationships that could be construed as a potential conflict of interest.

## Footnotes

^1^http://scholar.google.com.

^2^https://www.ncbi.nlm.nih.gov/pubmed.

^3^http://apps.webofknowledge.com/.
